# Advancing Cancer Research: Insights and Innovations in Pathogenesis and Targeted Therapies

**DOI:** 10.3390/jpm13030375

**Published:** 2023-02-21

**Authors:** Yong Wu, Jaydutt V. Vadgama

**Affiliations:** 1 Division of Cancer Research and Training, Department of Internal Medicine, Charles R. Drew University of Medicine and Science, Los Angeles, CA 90059, USA; 2 David Geffen UCLA School of Medicine, Los Angeles, CA 90095, USA; 3 UCLA Jonsson Comprehensive Cancer Center, Los Angeles, CA 90024, USA

Cancer is a global health challenge that continues to affect millions of people, despite extensive research efforts. The Special Issue on "Frontiers in Pathogenesis and Therapeutics of Cancer" presents a collection of research articles that aim to advance our understanding of cancer pathogenesis and develop targeted therapies to improve patient outcomes. In this Editorial, we provide a summary of the key insights and findings from these articles, highlighting their broader implications for cancer research.

The articles in this Special Issue address different aspects of cancer pathogenesis, such as the role of microRNAs in cancer progression and the influence of the tumor microenvironment on tumor growth and metastasis. They also explore the use of genetic and epigenetic markers for predicting cancer prognosis and developing diagnostic tools for early cancer detection. Additionally, several studies delve into the molecular mechanisms underlying cancer stem cell self-renewal and differentiation, as well as the development of targeted therapies that can selectively eliminate cancer stem cells.

Targeted therapies that selectively target cancer cells while sparing healthy cells have emerged as a promising approach for cancer treatment. This Special Issue highlights several innovative targeted therapies, including the use of engineered T cells to target cancer cells, the development of nanoparticles that can selectively deliver anticancer drugs to tumor cells, and the use of immunotherapy to activate the immune system against cancer. Moreover, precision medicine is another area of active research, with several studies focusing on developing personalized approaches for various types of cancer.

In conclusion, the articles in this Special Issue provide significant contributions to cancer research. They offer important insights into cancer pathogenesis and showcase novel approaches for cancer diagnosis and treatment. The development of targeted therapies and precision medicine approaches holds enormous potential to transform cancer treatment and improve patient outcomes. We hope that the findings presented in this Special Issue will inspire further research and ultimately lead to better cancer therapies and patient care.

